# A cost-effective barcode system for maize genetic discrimination based on bi-allelic InDel markers

**DOI:** 10.1186/s13007-020-00644-y

**Published:** 2020-07-29

**Authors:** Shuaiqiang Liang, Feng Lin, Yiliang Qian, Tifu Zhang, Yibo Wu, Yaocheng Qi, Sihai Ren, Long Ruan, Han Zhao

**Affiliations:** 1 Provincial Key Laboratory of Agrobiology, Institute of Crop Germplasm and Biotechnology, Jiangsu Academy of Agricultural Sciences, Nanjing, China; 2 Anhui Academy of Agricultural Sciences, Hefei, China

**Keywords:** Maize, Barcode system, InDel marker, Genetic discrimination

## Abstract

**Background:**

Maize is one of the most important cereal crop all over the world with a complex genome of about 2.3 gigabase, and exhibits tremendous phenotypic and molecular diversity among different germplasms. Along with the phenotype identification, molecular markers have been accepted extensively as an alternative tool to discriminate different genotypes.

**Results:**

By using previous re-sequencing data of 205 lines, bi-allelic insertions and deletions (InDels) all over maize genome were screened, and a barcode system was constructed consisting of 37 bi-allelic insertion-deletion markers with high polymorphism information content (PIC) values, large discriminative size among varieties. The barcode system was measured and determined, different maize hybrids and inbreds were clearly discriminated efficiently with these markers, and hybrids responding parents were accurately determined. Compared with microarray data of more than 200 maize lines, the barcode system can discriminate maize varieties with 1.57% of different loci as a threshold. The barcode system can be used in standardized easy and quick operation with very low cost and minimum equipment requirements.

**Conclusion:**

A barcode system was constructed for genetic discrimination of maize lines, including 37 InDel markers with high PIC values and user-friendly. The barcode system was measured and determined for efficient identification of maize lines.

## Background

The discrimination of plant variety and cultivar is one of the most important aspects in agricultural systems. Traditionally a variety is identified by a set of phenotype characteristics for official testing of distinctness, uniformity and stability (DUS). But due to various environmental and climatic conditions, the quantification of difference between varieties cannot be revealed precisely by these morphological descriptors, which is less suitable when results are required rapidly in large collections or breeding lines [1].

Molecular markers offer numerous advantages as they are stable and detectable in all tissues regardless of growth, differentiation, development, or defense status of the cell are not confounded by the environment, pleiotropic and epistatic effects [2]. Molecular markers have been widely used in genetic studies, marker-assisted selection, comparative mapping, and exploration of the functional genetic diversity in the germplasm adapted to different environments. The widely used molecular marker include RAPD (random amplified polymorphism DNA), SSR (simple sequence repeat), SNP (single nucleotide polymorphism), InDel (insertion-deletion) and so forth. Most of the markers have been used for cultivar identification. Through RAPD markers, efficient identification was performed for tomato, peach and Ribes cultivars [3–5]. By using EST-SSR markers, red-flesh loquat cultivars were rapidly identified [6]. SNP markers were used to genotype 260 accessions of Pummelo [7].

However, there are several disadvantages of the markers described above, for example, SSR markers are detected by polyacrylamide gel electrophoresis or capillary sequencing machines with small size differences, SNP identifications always depend on sequencing or microarray analysis. In contrast with SSRs and SNPs, InDel markers with moderate size differences of insertion-deletion polymorphisms are user-friendly, PCR-based with minimum equipment requirements, and co-dominant, offering more genomic information than SNPs [8–11] and have been widely used in population genetics, taxon diagnostic markers, genetic map construction and association mapping in different crop plants, such as rice [8], tomato [12], soybean [13], chickpea [14], capsicum [15], citrus [16] and so forth. Insertion-deletion polymorphisms in 3′ regions were used as highly informative genetic markers positioning corresponding expressed genes [17]. InDel markers were also developed for species identification [18]. According to InDel markers specific to dense variation blocks, a barcode system was constructed for Soybean identification [10]. Usually Insertion-deletion variances were multi-allelic and hampered genetic analysis since the segregation patterns of multi-allele are more complex and not appropriate for genome-wide analysis requiring large number of markers. With uncertainty of molecular weight, multi-allelic markers cannot be used in standardized operation. Another problem of multi-allele based analysis is prohibitively time-consuming computational speed with most large, genome-wide data sets. Genotypes of bi-allelic markers can be automated called by modern genotyping assays, suitable for massive data analysis [19]. In addition, bi-allelic markers producing simple differences are easily followed by different laboratories for both genetic research and plant breeding without molecular size calibrations.

Traditionally, it was difficult to automatically identify and genotype bi-allelic InDels due to less efficient sequencing technologies. By using an Affymetrix^®^ axiom^®^ array, InDels were high throughput genotyped in maize [20]. The development of next-generation sequencing (NGS) technology has paved the way for InDel identification. The massive amount of data and the short read nature of NGS created a hurdle for effective InDel variation mining, software have been developed for variant discovery, such as SAMtools [21], GATK [22] and Atlas2 [23]. A high-throughput and efficient pipeline was produced for genome-wide InDel marker development [11]. Based on whole genome re-sequencing, InDel markers were identified in Capsicum [24], Soybean [13], Quinoa [25], chickpea [14] etc. On the DNA sequence level, maize has a higher diversity level than humans, Drosophila and many wild plants [26, 27]. 30,178 indels were detected among elite maize inbred lines [28], facilitating the identification of InDel markers. By using next-generation sequencing data, genome-wide InDel markers were developed in maize [9].

In this study, bi-allelic InDel variations all over maize genome were screened by 205 re-sequenced genotypes, 8188 bi-allelic loci were identified and a barcode system consisting of 37 bi-allelic InDel markers with high PIC values and discriminative size larger than 20 bp which are suitable for agarose gel was constructed for genetic discrimination of maize inbred lines. By using these markers, different maize hybrids and inbreds were clearly discriminated efficiently, meanwhile, the corresponding parents of the hybrids were accurately determined.

## Methods

### Plant materials

To select proper InDel markers for barcode system, a total of 241 maize inbred lines (Additional file 1: Table S1) were used to test InDel primers, in which 227 lines were also analyzed by microarray. 177 intermated recombinant inbred lines (RILs) derived from B73 and Mo17 (the IBM population) and the parental lines were also employed to assess primers. 35 hybrid lines derived from 25 inbreds were used to evaluate the barcode system for pedigree analysis.

These materials were grown in the field at Nanjing, China in 2018, with 20 plants per row. All the plants were sampled at V4 stage, and three biological replicates per sample were harvested and mixed for DNA extraction.

### Genome sequence data and InDel marker development

The next generation sequence data of 205 maize inbred lines was downloaded from NCBI (Genbank accession number PRJNA82843, SRP011907 and PRJNA260788) [29–31]. After removing the low-quality nucleotides via SolexaQA with the Phred-Score greater than 20 [32], sequences of these materials were compared and those with the missing rate less than 10%, the minimum allele frequency (MAF) greater than 0.05 were selected. In addition, a linkage disequilibrium threshold (*r*^2^) of 0.20 with a window size of 100 and number of InDel to shift window at each step of 2 [33]. Linkage disequilibrium (LD) were measured by using the re-sequencing data with PLINK [33], the correlation coefficient (*r*^*2*^) of alleles were calculated by the software PopLDdecay [34].

The software mInDel was used for InDel marker development, InDel polymorphisms were identified using a sliding window alignment from assembled contigs with 300 bp of the window and 150 bp of the step, and dimorphic markers with large polymorphisms are preferred [11]. The loci of Insertion-deletions were annotated and predicted by SnpEff (version 4.3a) [35] based on maize B73 genome (version 4.32). Those sequences with polymorphic information content (PIC) greater than 0.4 were selected and analyzed with deep-depth sequencing data (30x) of six maize lines (Genbank accession number SRA010130) [28], the top 200 InDels were selected with the highest PIC for further analysis.

### PCR amplification and gel electrophoresis

Genomic DNA was extracted from young leaves following the method of plant DNA extraction kit from Qiagen. The PCR analysis was performed using 10 µL reaction mixtures containing 20 ng of genomic DNA, 2 pM of primer, and 5 µl of 2 × Taq Master Mix (Vazyme Biotech Co.,Ltd, China). PCR was performed under conditions of 95 °C for 3 min and subsequent 35 rounds of 94 °C for 30 s, 55 °C for 30 s, 72 °C for 30 s, and 72 °C 5 min. The PCR products were separated by electrophoresis in 2% gel of agarose followed by ethidium bromide staining. The cost of materials were listed in Additional file 2: Table S2, the total cost of PCR was 2.64 cents per sample. Usually the whole process took about 4 h for one sample.

### Barcoding process and identification of maize cultivars

Based on the selected InDels, the genetic distance between the maize lines were calculated with distance matrix and clustered using the UPGMA algorithm [36] in TASSEL 5.0 [37]. Phylogenetic tree was constructed by MEGA 6.0 [38].

### Genetic similarity analysis

227 maize inbred lines were genotyped by using the MaizeSNP50 (50 K) BeadChip based on Illumina platform as described by the manufacturer (Illumina, Inc. San Diego, CA). Then the genetic distance were calculated with the microarray data in Tassel 5.0 [37]. According to the InDel markers, the number of polymorphic loci between every pairs of lines were counted, together with the genetic distance, a boxplot was drawn by using PASW Statistics 18 (IBM SPSS).

## Results

### Insertion-deletion identification

To identify insertion-deletion variations in maize genome, the next generation sequence data of 205 maize inbred lines were used. Based on the genomic sequence comparison, 9,622,805 InDel variants were detected throughout the whole genome. Linkage disequilibrium throughout maize genome was measured and 11,741 LD blocks were obtained by using PLINK. After removing those with missing rate > 10%, MAF < 0.05, and PIC < 0.4, 25,412 insert-deletion variants located in the LD blocks were identified. Only one insert-deletion polymorphism was kept for each block, and 11,741 InDels were used for further analysis.

To facilitate screening using gel-electrophoresis, only InDels larger than 20 bp in length and bi-allelic polymorphism loci were selected and 8188 InDels were used for further analysis. These InDels distributed across all maize genome. A maximum of InDels (1252) were identified on chromosome 1 while the fewest (548) were detected on chromosome 10. Annotation of these variants showed 11 genomic locations, including UTR_3_prime, UTR_5_prime, downstream, intergenic, intron, exon, upstream and so forth (Table 1). The maximum amount of 2622 InDels (32.02%) were located in the intergenic region, the second amount of 2207 (26.95%) were assigned at the upstream region. Another two locations exist more than 1000 InDels, including downstream, and intron (Table 1).

**Table 1:** Annotation of 8188 bi-allelic polymorphism insertion deletion loci detected across maize genome

InDel annotation	Number of InDels	Percentage
UTR_3_Prime	371	4.53%
UTR_5_Prime	210	2.56%
Transcript	2	0.02%
Downstream	1482	18.10%
Intergenic	2622	32.02%
Intron	1029	12.57%
Splice_site_acceptor	1	0.01%
Splice_site_donor	3	0.04%
Splice_site_region	55	0.67%
Exon	206	2.52%
Upstream	2207	26.95%

### Validation of InDel markers for cultivar discrimination

**Fig. 1:**
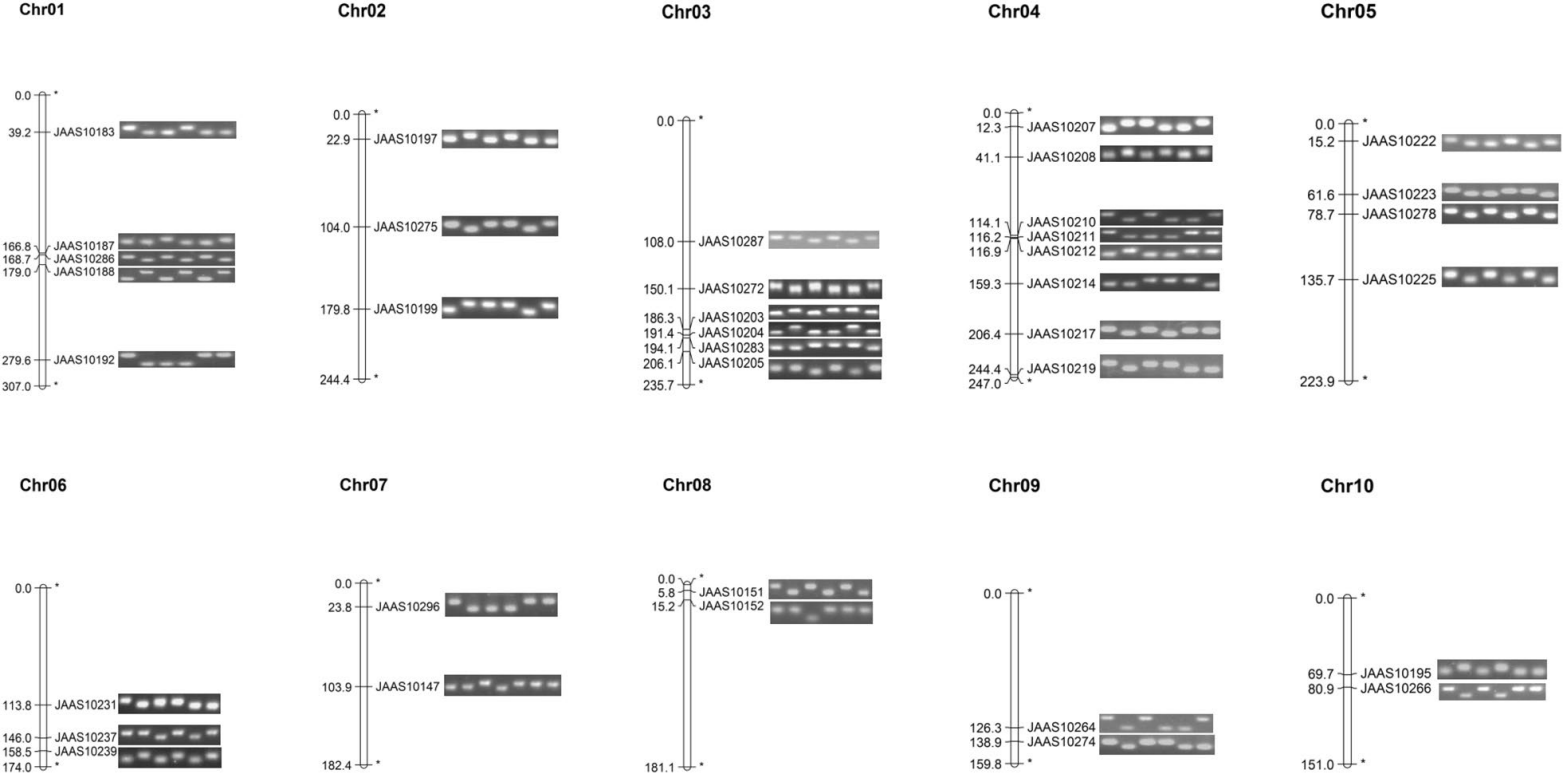
The chromosome location of 37 InDel markers developed for barcode system

**Fig. 2:**
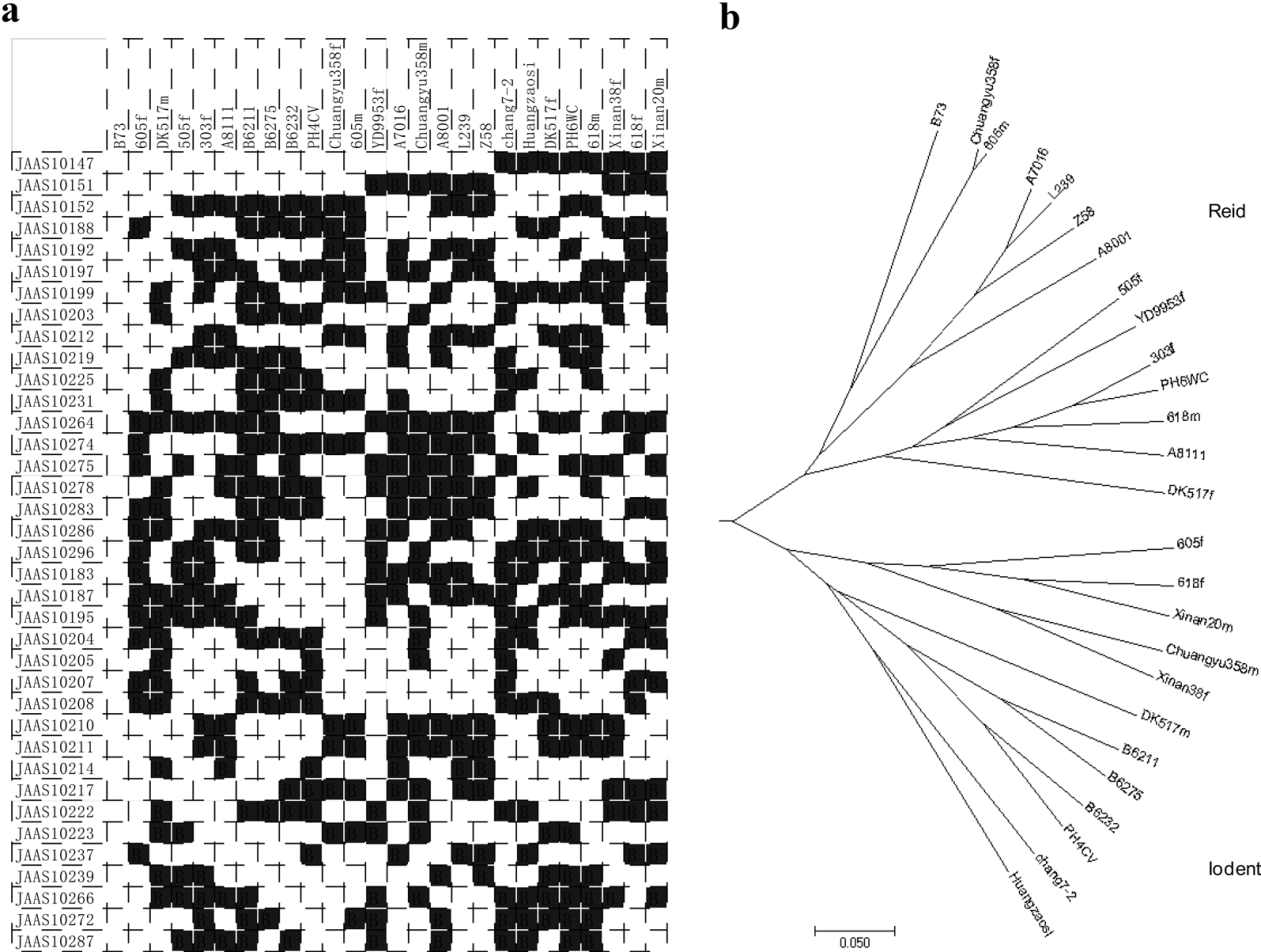
Barcode system based on 37 InDel markers and evaluated in 26 inbred maize lines. **a** Barcoding representation of the polymorphisms revealed by the InDel markers among 26 maize lines, the same allele with B73 were represented by “A” depicted as “white” barcode, and the alternative allele was designed to “B” depicted as “black” barcode, respectively; **b** Phylogenetic tree constructed with 37 InDels in 26 maize lines

Primers for 200 InDels were designed and tested by using 177 lines of IBM population. Based on the electrophoresis with 2% agarose gel, those primer sets with clear band and significant difference among the 177 lines were performed segregation analysis in the IBM population. 37 primer sets were selected for barcoding system (Table 2). They are distributed on all ten chromosomes of maize, with at least two markers per chromosome (Fig. 1). To evaluate the discriminating ability of the InDel markers, 26 inbred lines were genotyped with them. According to the electrophoresis results of the 37 markers, all the maize lines were discriminated by at least one locus (Fig. 2a), implying suitable ability for maize cultivar discrimination. Since InDels were selected for bi-allelic polymorphism loci, primers would produce two types of amplicons, insertion or deletion, relative to the reference genome. Based on the amplification results, the same allele with B73 were represented by “A” depicted as “white” barcode, and the alternative allele was designed to “B” depicted as “black” barcode, respectively (Fig. 2a). Phylogenetic tree was drawn based on the genotypes, showing that these maize lines were separated into two major groups (Fig. 2b), corresponding to Reid and Iodent heterotic groups, respectively.

**Table 2:** 37 InDel markers selected for barcode system

Primers	Chr	B73 start	B73 end
JAAS10183	1	39,220,755	39,220,901
JAAS10187	1	166,757,275	166,757,431
JAAS10286	1	168,743,620	168,743,826
JAAS10188	1	178,998,629	178,998,955
JAAS10192	1	279,588,335	279,588,611
JAAS10197	2	22,940,391	22,940,126
JAAS10275	2	103,989,933	103,990,132
JAAS10199	2	179,799,703	179,799,946
JAAS10287	3	108,017,920	108,017,718
JAAS10272	3	150,110,674	150,110,880
JAAS10203	3	186,344,389	186,344,632
JAAS10204	3	191,420,611	191,420,850
JAAS10283	3	194,095,825	194,096,026
JAAS10205	3	206,096,973	206,097,093
JAAS10207	4	12,274,984	12,275,169
JAAS10208	4	41,061,935	41,062,041
JAAS10210	4	114,051,646	114,051,820
JAAS10211	4	116,167,826	116,167,966
JAAS10212	4	116,878,227	116,878,377
JAAS10214	4	159,347,188	159,347,336
JAAS10217	4	206,411,354	206,411,479
JAAS10219	4	244,411,309	244,411,561
JAAS10222	5	15,179,127	15,179,275
JAAS10223	5	61,550,502	61,550,667
JAAS10278	5	78,678,360	78,678,565
JAAS10225	5	135,676,963	135,677,087
JAAS10231	6	113,761,684	113,761,858
JAAS10237	6	146,027,298	146,027,511
JAAS10239	6	158,527,019	158,527,123
JAAS10296	7	23,765,684	23,765,919
JAAS10147	7	103,865,590	103,865,768
JAAS10151	8	5,790,426	5,790,775
JAAS10152	8	15,227,845	15,228,052
JAAS10264	9	126,272,395	126,272,596
JAAS10274	9	138,866,336	138,866,540
JAAS10195	10	69,676,147	69,676,254
JAAS10266	10	80,881,811	80,882,025

### Evaluation of the barcode system for pedigree tracing

The maize barcode system was evaluated with 35 hybrids derived from 25 inbreds. Theoretically 25 inbred lines can produce 300 hybrids without regard to reciprocal cross. According to genotypes of the parents by using the 37 InDel markers, genotypes of all 300 descendants were predicted. The barcode of 35 randomly selected hybrid lines were genotyped based on the InDels and compared with the 300 predicted genotype data. Among the 37 loci invested, the loci with equal experimental results and predicted data were counted and the largest number of matched loci suggested the most possibility of correct prediction. Table 3 showed top two matched loci number with equal experimental and predicted results. In the top one column, the matched loci number ranged from 27 to 37, all higher than that in the second column (Table 3). Together with the combination data of maize hybrids, all the prediction data with top one matched loci were correct, confirming that the barcode system was suitable for pedigree tracing analysis.

**Table 3:** Pedigree analysis with experimental and predicted results of 35 hybrids based on the 37 InDel barcode system

Hybrids to be tested	Predicted pedigree 1	Matched loci number	Predicted pedigree 2	Matched loci number
hyb1	A7016 × B6211	34	L239 × B6211	31
hyb2	A7016 × B6232	36	L239 × B6232	33
hyb3	A7016 × DK517m	34	L239 × DK517m	31
hyb4	A7016 × Xinan20m	37	L239 × Xinan20m	34
hyb5	A8001 × B6211	32	303f × B6275	28
hyb6	A8111 × B6211	32	A8111 × B6275	28
hyb7	A8111 × DK517m	30	618 m × DK517m	27
hyb8	B6211 × Chuangyu358m	34	B6275 × Chuangyu358m	30
hyb9	DK517f × A7016	29	DK517f × L239	26
hyb10	DK517f × B6211	35	DK517f × B6275	31
hyb11	DK517f × B6232	37	DK517f × PH4CV	31
hyb12	DK517f × PH4CV	35	DK517f × B6232	31
hyb13	DK517f × Xinan20m	36	618f × DK517f	29
hyb14	L239 × DK517m	27	Z58 × DK517m	26
hyb15	L239 × Xinan20m	36	Z58 × Xinan20m	34
hyb16	PH6WC × B6211	33	303f × B6211	30
hyb17	PH6WC × PH4CV	33	303f × PH4CV	29
hyb18	303f × B6211	35	PH6WC × B6211	32
hyb19	303f × PH4CV	35	PH6WC × PH4CV	33
hyb20	303f × Xinan20m	36	PH6WC × Xinan20m	32
hyb21	505f × B6211	35	505f × B6275	31
hyb22	505f × B6232	37	505f × PH4CV	31
hyb23	505f × PH4CV	35	505f × B6232	31
hyb24	505f × Xinan20m	36	618f × X505f	31
hyb25	605f × B6211	34	605f × B6275	30
hyb26	605f × PH4CV	34	605f × B6232	30
hyb27	618f × DK517f	27	618f × DK517m	26
hyb28	618f × DK517f	31	618f × PH6WC	28
hyb29	YD9953f × DK517m	29	YD9953f × Chang7-2	24
hyb30	YD9953f × Chuangyu358f	35	YD9953f × 605 m	34
hyb31	YD9953f × Xinan20m	34	618f × YD9953f	30
hyb32	Z58 × B6211	36	L239 × B6211	32
hyb33	DK517m × Chuangyu358m	29	YD9953f × DK517m	27
hyb34	Chuangyu358m × PH4CV	34	B6232 × Chuangyu358m	30
hyb35	Xinan38f × B6211	34	Xinan38f × B6275	30

### Database construction with the barcode system in a maize population

**Fig. 3:**
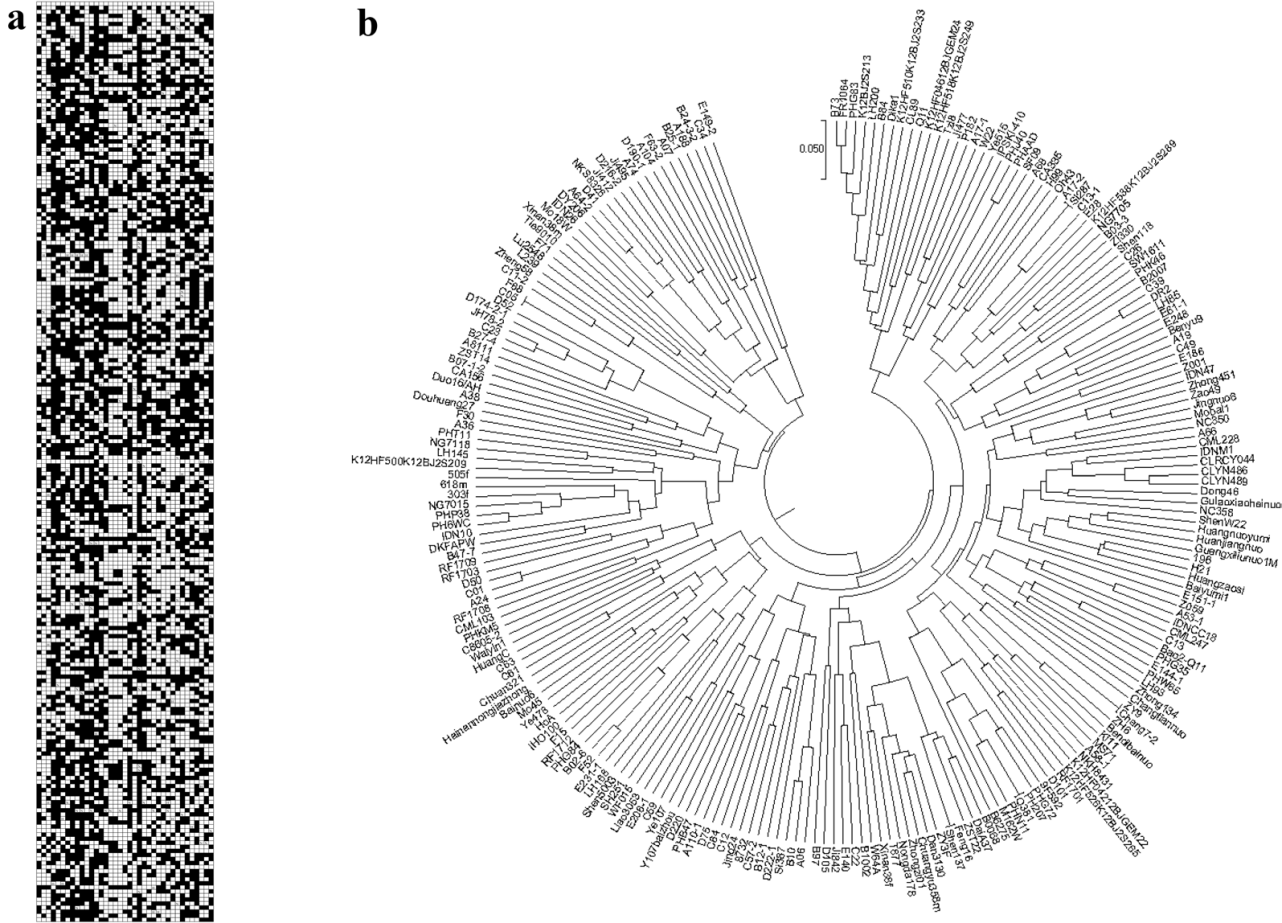
Database with barcode system in maize population including 227 maize lines. **a** Barcoding among 227 maize lines, the same allele with B73 were represented by “A” depicted as “white” barcode, and the alternative allele was designed to “B” depicted as “black” barcode, respectively; **b** Phylogenetic tree drawn with 37 InDels in 227 maize lines

A population including 227 lines was used for database construction with the barcode InDel markers (Fig. 3a, Additional file 3: Table S3). A total of 8399 genotype data were recorded in the database with only 75 missing data, accounting for 99.1% of data integrity. Among the 227 inbred lines, 56 hybrid loci (0.66%) were detected, implying most of these materials were highly homozygous. In the population, PIC of the 37 InDels ranged from 0.2910 to 0.4998, in which only two less than 0.35 and 30 larger than 0.40.

Based on the barcode, more than 99.98% of the material pairs were discriminated with at least two InDel markers. The number of polymorphic loci detected by the InDels markers between each cultivar pairs ranged from zero to 34, with the average 17 (Additional file 4: Table S4). Among all 25,651 maize line pairs, five pairs showed no difference by using the 37 InDel markers and assigned at the same location on the phylogenetic tree (Fig. 3b, Additional file 4: Table S4). The population was also genotyped with microarray analysis, consisting of 55,187 loci. On the phylogenetic tree drawn with microarray data, the five pairs of maize lines were also located at the same places (Additional file 5: Fig. S1) with 1027(A17-2 vs Si287), 164 (C05 vs F68), 525 (PH207 vs Q381), 1014 (ZY9 vs Chang7-2), and 1386 (Feng16 vs Shen137) polymorphic SNPs, respectively, showing the close relationship between these pairs of lines. Based on the microarray data, the average number of different loci between the five pairs of lines was 823, accounting for 1.57% of the total loci. With the barcode system, all the other materials showed differences at least one locus and the minimum average number of different loci was 3056 according to microarray data, significantly different (*p *= 0.013) from that of the five pairs of lines with no difference based on the barcode system. The result suggested the threshold was 1.57% of different loci which can be discriminated with our barcode system.

**Fig. 4:**
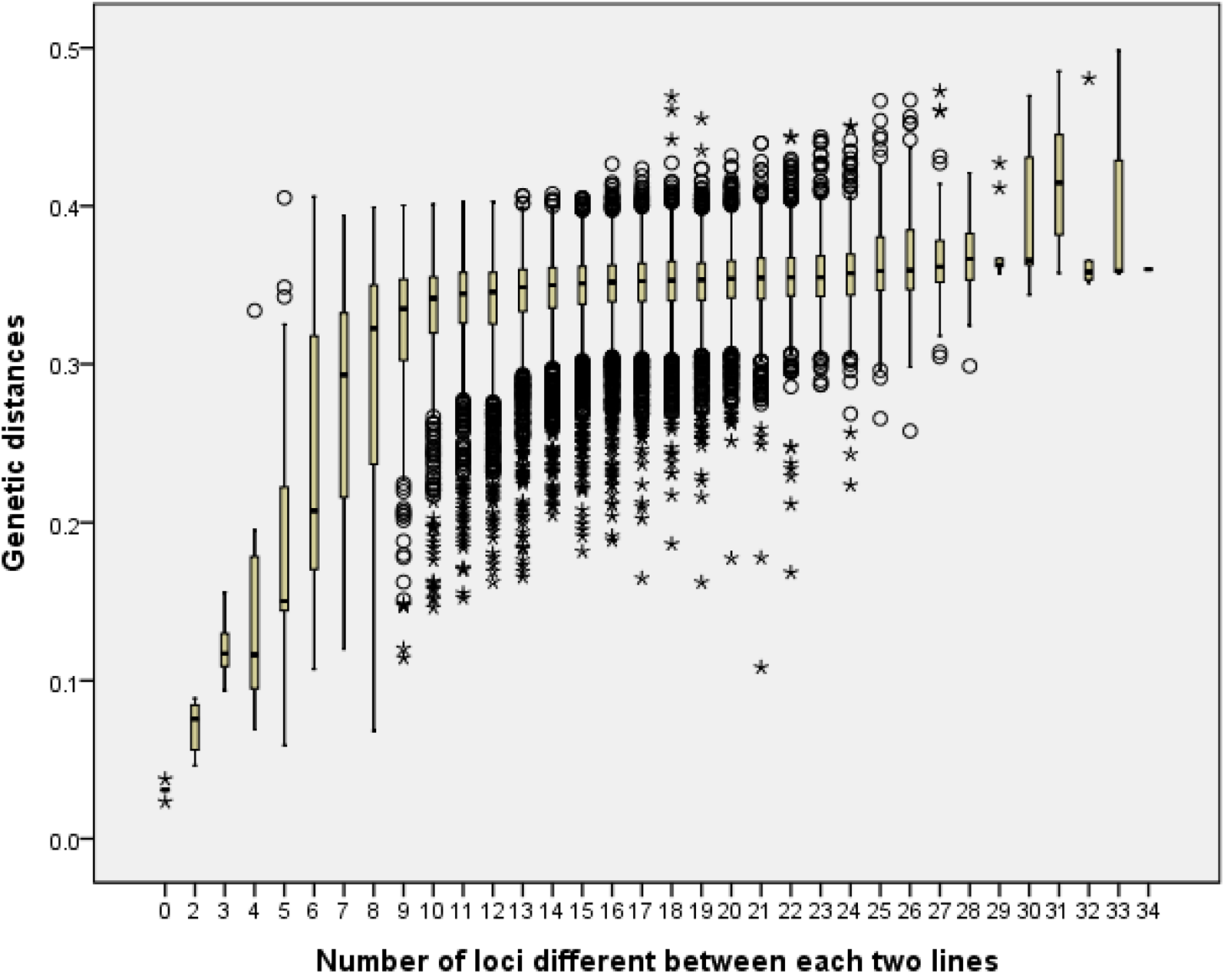
The genetic distances according to the number of loci different between each two maize lines. The genetic distances were calculated by using the microarray data of each two maize lines

By using the microarray data, genetic distances of each two lines were calculated. The lowest genetic distance based on the SNP data were detected between the five pairs of maize lines with no different according to InDel markers. The genetic distances increased rapidly along with the number of polymorphic loci until became steady when the number of polymorphic InDels reach ten (Fig. 4), implying ten InDel markers can reveal most of the genetic variance among the population with the average genetic distance of 0.33.

## Discussion

Maize is one of the most important cereal crop throughout the world with the highest yield. More and more cultivars were produced for the market. Then identification of maize variety and cultivar become more important than ever before with profound meanings to ensure seed quality and food safety [39]. Along with the phenotype identification, such as DUS, molecular marker has been widely used for cultivar identification due to numerous advantages. In this study, we produced a barcode system for maize cultivars identification with bi-allelic InDel markers based on next generation sequencing data with several advantages such as high discrimination ability, standardization, low-cost, easy and quick operation.

Molecular markers have been used for accurate and precise discrimination of cultivars, such as SNP markers [40], and microsatellite markers [41]. In China, SSR-based standard fingerprint database was constructed for corn variety authorization [42]. Insertion-deletion are structural variations distributed throughout the genome, sometimes lead to the gain/loss of function in the organism [43]. In contrast with SSR and SNP markers, InDel markers were used to determine genetic variation with the merit of easy detection of polymorphisms by PCR and direct gel electrophoresis. In rice InDel markers were developed to discriminate genome types rapidly [44].

The progress of sequencing technologies has paved the way for understanding the plant genome and more and more lines have been sequenced. The massive data has helped researchers to genetically characterize the genomes and screen InDel loci. By aligning the B73 and Mo17 genomes, 1,422,446 small insertions/deletions (length shorter than 100 bp) were identified in maize [45]. Based on next-generation sequencing data, genome-wide InDel markers have been discovered in Chickpea [14], Quinoa [25], Soybean [13], Brassica [46], Capsicum [15] and so forth. In maize, genome-wide insertion and deletion markers were also developed [9]. According to the next generation sequence data of 205 maize inbred lines, 9,622,805 InDel variants were detected throughout the whole genome in this study.

With the abundant insertion-deletion variants, those were selected for barcode system based on several criteria, including convenient for detect and analyze, and high discrimination ability. In order to separate amplicons appropriately on agarose gels, InDels longer than 20 bp were selected. Recently, more and more researches have to deal with massive data and multiple allelic loci hampered automatic analysis with computers. Bi-allelic loci conquered this problem, and genotypes of bi-allelic markers are more suitable for automatic analysis [19]. With pan-genome sequence data, InDels at the bi-allelic loci were developed in this study. PIC (polymorphism information content) was another factor used for marker selection. According to the next generation sequence data, the InDel markers for barcode system in this study were dimorphic polymorphisms with higher PIC and can be resolved appropriately by electrophoresis and user-friendly in a standardized operation. Another point of our barcode system is the low cost, less than 1 dollar per sample for analysis with all barcode makers, suitable for plant breeding with large scale screen. Typically the whole process took about 4 h for one sample, while much less time was taken for batch operation. For example, 2 h electrophoresis could run 200 samples at one time.

Authentication of plant species is important in a variety of different areas such as the trade of illegal and endangered species and food authentication [47]. DNA barcoding is a technique for characterizing species of organisms using a short DNA sequence from a standard and agreed-upon position in the genome and has sufficient sequence variation to discriminate among species [48]. The main purpose of barcode system is to discriminate different cultivars in an efficient way. Usually several leading candidate barcodes were used for plant DNA barcoding, including *rbcL*, *rpoB*, *rpoC1*, *matK*, *atpF*-*atpH*, *psbK*-*psbI* and *trnH*-*psbA* [49]. However, due to differences in their efficiency, it was concluded that no single-locus plant barcode exists [50]. In this study, based on genome-wide screen, a barcode system with InDel markers were constructed in maize. With high throughput sequence data, barcode candidates at the conserved regions among different lines were detected through the genome variances of 205 maize lines. Their ability of cultivar identification were measured experimentally. Both inbred lines and hybrids were used to test these InDel markers. By using 26 inbred lines, 37 InDel markers could discriminate all of them by at least one locus (Fig. 2). Based on the discrimination ability, the barcode system was evaluated for their use in pedigree tracing. 35 hybrid descendants from 25 inbreds were tested by using the barcode system, 27 to 37 markers made the correct prediction for their pedigree, confirming their ability for identification.

To test the accuracy of their discrimination, a population including 227 lines were genotyped by both the barcode and DNA microarray. Among all 227 lines, only five pairs of lines was not detected difference by using the 37 InDel markers. According to the genetic distances compared with microarray data including more than 50 k loci, the InDel can reveal the genetic variance effectively, with the threshold of 1.57% of different loci. Although all 37 markers used for identification was more efficiency, when the InDel variances above ten, the genetic distance kept steady (Fig. 4).

However, there were still several limits for the barcode system. Those materials with lower difference than the threshold cannot be discriminated by the system, for instance, sib-lines, backcross improved lines, mutants etc. And these markers were selected with several criterions and not suitable for genomic prediction due to the low number of markers. We are working to develop efficient and low-cost markers for genome prediction.

## Conclusions

This study constructed a barcode system for genetic discrimination of maize lines by using re-sequencing data of 205 lines, including 37 bi-allelic InDel markers with high PIC values and user-friendly. The barcode system was measured and determined and different maize hybrids and inbreds were clearly discriminated efficiently with these markers, and the corresponding parental lines of the hybrids were accurately determined. The barcode system can be used in standardized easy and quick operation with very low cost and minimum equipment requirements.

## Supplementary information

**Additional file 1: Table** **1.** The list of 241 maize inbred lines used in the study.

**Additional file 2: Table** **2.** The cost of materials used in PCR for one sample.

**Additional file 3: Table** **3.** The database constructed with the 37 barcode InDel markers in 227 maize lines.

**Additional file 4: Table** **4.** The number of different loci discriminated by the 37 barcode InDel markers among 227 maize lines.

**Additional file 5: Figure S1**. Phylogenetic tree constructed with the 37 InDels in 227 maize lines.

## Data Availability

The datasets used and/or analyzed during the current study are available from the corresponding author on reasonable request.

## References

[1] Korir NK, Han J, Shangguan L, Wang C, Kayesh E, Zhang Y, Fang J (2013). Plant variety and cultivar identification: advances and prospects. Crit Rev Biotechnol.

[2] Agarwal M, Shrivastava N, Padh H (2008). Advances in molecular marker techniques and their applications in plant sciences. Plant Cell Rep.

[3] Huo J, Yang G, Zhang Y, Li F (2013). A new strategy for identification of currant (Ribes nigrum L) cultivars using RAPD markers. Genet Mol Res.

[4] Han J, Wang WY, Leng XP, Guo L, Yu ML, Jiang WB, Ma RJ (2014). Efficient identification of ornamental peach cultivars using RAPD markers with a manual cultivar identification diagram strategy. Genet Mol Res.

[5] Korir NK, Li XY, Leng XP, Wu Z, Wang C, Fang JG (2013). A novel and efficient strategy for practical identification of tomato (Solanum lycopersicon) varieties using modified RAPD fingerprints. Genet Mol Res.

[6] Li XY, Xu HX, Chen JW (2014). Rapid identification of red-flesh loquat cultivars using EST-SSR markers based on manual cultivar identification diagram strategy. Genet Mol Res.

[7] Wu B, Zhong GY, Yue JQ, Yang RT, Li C, Li YJ, Zhong Y, Wang X, Jiang B, Zeng JW (2014). Identification of pummelo cultivars by using a panel of 25 selected SNPs and 12 DNA segments. PLoS ONE.

[8] Wu DH, Wu HP, Wang CS, Tseng HY, Hwu KK (2013). Genome-wide InDel marker system for application in rice breeding and mapping studies. Euphytica.

[9] Liu J, Qu J, Yang C, Tang D, Li J, Lan H, Rong T (2015). Development of genome-wide insertion and deletion markers for maize, based on next-generation sequencing data. BMC Genomics.

[10] Sohn HB, Kim SJ, Hwang TY, Park HM, Lee YY, Markkandan K, Lee D, Lee S, Hong SY, Song YH (2017). Barcode System for Genetic Identification of Soybean [Glycine max (L.) Merrill] Cultivars Using InDel Markers Specific to Dense Variation Blocks. Front Plant Sci.

[11] Lv Y, Liu Y, Zhao H (2016). mInDel: a high-throughput and efficient pipeline for genome-wide InDel marker development. BMC Genomics.

[12] Jin L, Zhao L, Wang Y, Zhou R, Song L, Xu L, Cui X, Li R, Yu W, Zhao T: Genetic diversity of 324 cultivated tomato germplasm resources using agronomic traits and InDel markers. *Euphytica* 2019, 215(4).

[13] Song X, Wei H, Cheng W, Yang S, Zhao Y, Li X, Luo D, Zhang H, Feng X (2015). Development of INDEL markers for genetic mapping based on whole genome resequencing in soybean. Genes Genomes Genetics.

[14] Das S, Upadhyaya HD, Srivastava R, Bajaj D, Gowda CL, Sharma S, Singh S, Tyagi AK, Parida SK (2015). Genome-wide insertion-deletion (InDel) marker discovery and genotyping for genomics-assisted breeding applications in chickpea. DNA Res.

[15] Guo G, Zhang G, Pan B, Diao W, Liu J, Ge W, Gao C, Zhang Y, Jiang C, Wang S: Development and Application of InDel Markers for Capsicum spp. Based on Whole-Genome Re-Sequencing. *Scientific Reports* 2019, 9(1). 10.1038/s41598-019-40244-y PMC6403297 30842649

[16] Fang Q, Wang L, Yu H, Huang Y, Jiang X, Deng X, Xu Q (2018). Development of Species-Specific InDel Markers in Citrus. Plant Mol Biol Rep.

[17] Bhattramakki D, Dolan M, Hanafey M, Wineland R, Vaske D, Register JC, Tingey SV, Rafalski A (2002). Insertion-deletion polymorphisms in 3′ regions of maize genes occur frequently and can be used as highly informative genetic markers. Plant Mol Biol.

[18] Park I, Yang S, Kim WJ, Song JH, Lee HS, Lee HO, Lee JH, Ahn SN, Moon BC: Sequencing and Comparative Analysis of the Chloroplast Genome of Angelica polymorpha and the Development of a Novel Indel Marker for Species Identification. *Molecules* 2019, 24(6). 10.3390/molecules24061038 PMC6471784 30875988

[19] Voorrips RE, Gort G, Vosman B (2011). Genotype calling in tetraploid species from bi-allelic marker data using mixture models. BMC Bioinform.

[20] Mabire C, Duarte J, Darracq A, Pirani A, Rimbert H, Madur D, Combes V, Vitte C, Praud S, Riviere N (2019). High throughput genotyping of structural variations in a complex plant genome using an original Affymetrix(R) axiom(R) array. BMC Genomics.

[21] Li H, Handsaker B, Wysoker A, Fennell T, Ruan J, Homer N, Marth G, Abecasis G, Durbin R (2009). The Sequence Alignment/Map format and SAMtools. Bioinformatics.

[22] McKenna A, Hanna M, Banks E, Sivachenko A, Cibulskis K, Kernytsky A, Garimella K, Altshuler D, Gabriel S, Daly M (2010). The Genome Analysis Toolkit: a MapReduce framework for analyzing next-generation DNA sequencing data. Genome Res.

[23] Challis D, Yu J, Evani US, Jackson AR, Paithankar S, Coarfa C, Milosavljevic A, Gibbs RA, Yu F (2012). An integrative variant analysis suite for whole exome next-generation sequencing data. BMC Bioinform.

[24] Karna S, Ahn Y-K (2018). Development of InDel markers to identify Capsicum disease resistance using whole genome resequencing. J Plant Biotechnol.

[25] Zhang T, Gu M, Liu Y, Lv Y, Zhou L, Lu H, Liang S, Bao H, Zhao H (2017). Development of novel InDel markers and genetic diversity in Chenopodium quinoa through whole-genome re-sequencing. BMC Genomics.

[26] Tenaillon MI, Sawkins MC, Long AD, Gaut RL, Doebley JF, Gaut BS (2001). Patterns of DNA sequence polymorphism along chromosome 1 of maize (Zea mays ssp. mays L.). Proc Natl Acad Sci U S A..

[27] Wright SI, Gaut BS (2005). Molecular population genetics and the search for adaptive evolution in plants. Mol Biol Evol.

[28] Lai J, Li R, Xu X, Jin W, Xu M, Zhao H, Xiang Z, Song W, Ying K, Zhang M (2010). Genome-wide patterns of genetic variation among elite maize inbred lines. Nat Genet.

[29] Jiao Y, Zhao H, Ren L, Song W, Zeng B, Guo J, Wang B, Liu Z, Chen J, Li W (2012). Genome-wide genetic changes during modern breeding of maize. Nat Genet.

[30] Unterseer S, Bauer E, Haberer G, Seidel M, Knaak C, Ouzunova M, Meitinger T, Strom TM, Fries R, Pausch H (2014). A powerful tool for genome analysis in maize: development and evaluation of the high density 600 k SNP genotyping array. BMC Genomics.

[31] Chia J-M, Song C, Bradbury PJ, Costich D, de Leon N, Doebley J, Elshire RJ, Gaut B, Geller L, Glaubitz JC (2012). Maize HapMap2 identifies extant variation from a genome in flux. Nat Genet.

[32] Cox MP, Peterson DA, Biggs PJ (2010). SolexaQA: at-a-glance quality assessment of Illumina second-generation sequencing data. BMC Bioinform.

[33] Purcell S, Neale B, Todd-Brown K, Thomas L, Ferreira MAR, Bender D, Maller J, Sklar P, de Bakker PIW, Daly MJ (2007). PLINK: a tool set for whole-genome association and population-based linkage analyses. Am J Human Genet.

[34] Zhang C, Dong SS, Xu JY, He WM, Yang TL: PopLDdecay: a fast and effective tool for linkage disequilibrium decay analysis based on variant call format files. *Bioinformatics* 2018. 10.1093/bioinformatics/bty875 30321304

[35] Cingolani P, Platts A, le Wang L, Coon M, Nguyen T, Wang L, Land SJ, Lu X, Ruden DM (2012). A program for annotating and predicting the effects of single nucleotide polymorphisms, SnpEff: sNPs in the genome of Drosophila melanogaster strain w1118; iso-2; iso-3. Fly (Austin).

[36] Saitou N, Nei M (1987). The neighbor-joining method: a new method for reconstructing phylogenetic trees. Mol Biol Evol.

[37] Bradbury PJ, Zhang Z, Kroon DE, Casstevens TM, Ramdoss Y, Buckler ES (2007). TASSEL: software for association mapping of complex traits in diverse samples. Bioinformatics.

[38] Kumar S, Stecher G, Tamura K (2016). MEGA7: molecular evolutionary genetics analysis version for bigger datasets. Mol Biol Evol.

[39] Wang FG, Tian HL, Yi HY, Zhao H, Huo YX, Kuang M, Zhang LK, Lv YD, Ding MQ, Zhao JR (2018). Principle and strategy of DNA fingerprint identification of plant variety. Mol Plant Breed.

[40] Liu W, Xiao Z, Bao X, Yang X, Fang J, Xiang X (2015). Identifying litchi (Litchi chinensis Sonn.) cultivars and their genetic relationships using single nucleotide polymorphism (SNP) markers. PLoS ONE.

[41] Sousa TV, Caixeta ET, Alkimim ER, de Oliveira ACB, Pereira AA, Zambolim L, Sakiyama NS: Molecular markers useful to discriminate Coffea arabica cultivars with high genetic similarity. *Euphytica* 2017, 213(3).

[42] Wang FG, Yang Y, Yi HM, Zhao JR, Ren J, Wang L, Ge JR, Jiang B, Zhang XC, Tian HL, Hou ZH (2017). Construction of an SSR-based standard fingerprint database for corn variety authorized in China. Scientia Agricultura Sinica.

[43] Jain A, Roorkiwal M, Kale S, Garg V, Yadala R, Varshney RK (2019). InDel markers: an extended marker resource for molecular breeding in chickpea. PLoS ONE.

[44] Yamaki S, Ohyanagi H, Yamasaki M, Eiguchi M, Miyabayashi T, Kubo T, Kurata N, Nonomura KI (2013). Development of INDEL markers to discriminate all genome types rapidly in the genus Oryza. Breed Sci.

[45] Sun S, Zhou Y, Chen J, Shi J, Zhao H, Zhao H, Song W, Zhang M, Cui Y, Dong X (2018). Extensive intraspecific gene order and gene structural variations between Mo17 and other maize genomes. Nat Genet.

[46] Liu B, Wang Y, Zhai W, Deng J, Wang H, Cui Y, Cheng F, Wang X, Wu J (2013). Development of InDel markers for Brassica rapa based on whole-genome re-sequencing. Theor Appl Gene.

[47] Ballin NZ, Onaindia JO, Jawad H, Fernandez-Carazo R, Maquet A (2019). High-resolution melting of multiple barcode amplicons for plant species authentication. Food Control.

[48] Li X, Yang Y, Henry RJ, Rossetto M, Wang Y, Chen S (2015). Plant DNA barcoding: from gene to genome. Biol Rev Camb Philos Soc.

[49] Hollingsworth PM, Forrest LL, Spouge JL, Hajibabaei M, Ratnasingham S, van der Bank M, Chase MW, Cowan RS, Erickson DL, Fazekas AJ (2009). A DNA barcode for land plants. Proc Natl Acad Sci.

[50] Tnah LH, Lee SL, Tan AL, Lee CT, Ng KKS, Ng CH, Nurul Farhanah Z (2019). DNA barcode database of common herbal plants in the tropics: a resource for herbal product authentication. Food Control.

