# Supplementary figures and images for: A cost-effective barcode system for maize genetic discrimination based on bi-allelic InDel markers
Source: Plant Methods. 2020 Jul 29;16:101. doi: 10.1186/s13007-020-00644-y (PMC7391534; doi:10.1186/s13007-020-00644-y)

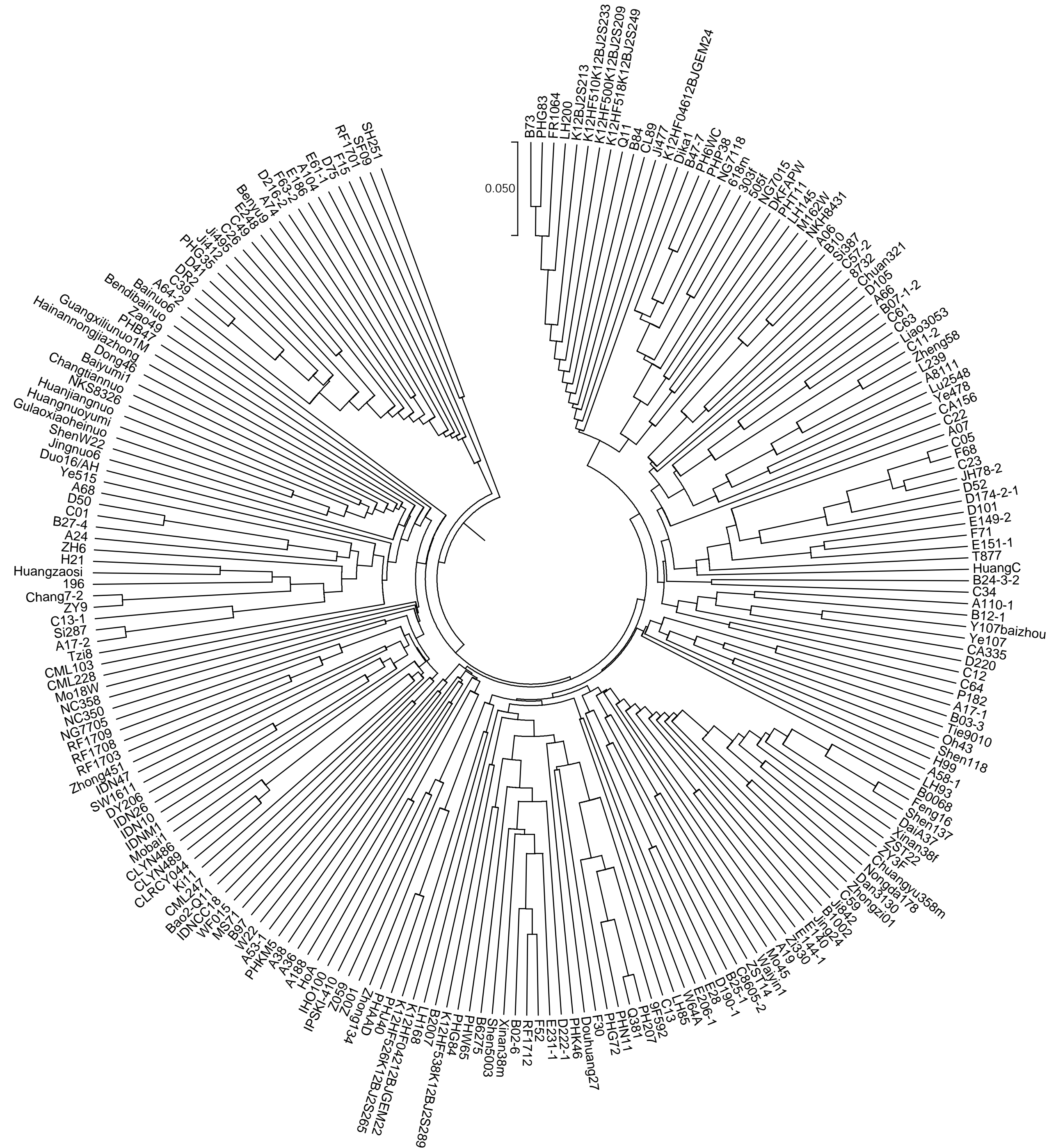

Supplement: Supplementary file 5 — Additional file 5: Figure S1. Phylogenetic tree constructed with the 37 InDels in 227 maize lines. [file 13007_2020_644_MOESM5_ESM.pdf]
